# First Description of a Carnivore Protoparvovirus Associated with a Clinical Case in the Iberian Lynx (*Lynx pardinus*)

**DOI:** 10.3390/ani15071026

**Published:** 2025-04-02

**Authors:** Almudena Campoy, Esperanza Gomez-Lucia, Tania Garcia, Elena Crespo, Sonia Olmeda, Felix Valcarcel, Sergio Fandiño, Ana Domenech

**Affiliations:** 1 Department of Animal Health, Faculty of Veterinary Medicine, Complutense University of Madrid, 28040 Madrid, Spain; almuvet29@gmail.com (A.C.); duato@ucm.es (E.G.-L.); tania_tkd_98@hotmail.com (T.G.); angeles@ucm.es (S.O.); sergifan@ucm.es (S.F.); 2 Research Group of “Animal Viruses”, Complutense University of Madrid, 28040 Madrid, Spain; 3 Wildlife Recovery Centre “El Chaparrillo”, 13071 Ciudad Real, Castilla-La-Mancha, Spain; elena.crespo@externas.jccm.es; 4 Group of Animal Parasitology, Department of Animal Reproduction, INIA-CSIC, 28040 Madrid, Spain; valcarcel.felix@inia.csic.es

**Keywords:** Iberian lynx, parvovirus, faeces, PCR, complete sequence, FPV

## Abstract

One of the main threats for the survival of the Iberian lynx is infectious disease. Feline parvoviruses cause often fatal diseases in cats and have been isolated from different species of Felidae and other carnivores. The present study is the first description of a parvoviral sequence isolated from the brain of an Iberian lynx which died four weeks after being transferred to a quarantine centre from a hunting estate in Castilla-La-Mancha (southern border of the Iberian plateau). Four days prior to death, it had developed anorexia and muscle weakness. The nucleotide sequence, at 4589 nt long (GenBank PP781551), was most proximal to that isolated from a Eurasian badger in Italy but also showed great homology with others from cats and other carnivores isolated in Spain and Italy, including that from a cat sequenced by us to elucidate the origin of the infection, which has not been clarified. The phylogenetic analysis of the capsid protein, VP2, which determines tropism and host range, confirmed that the lynx sequence was most proximal to feline than to canine parvoviruses, and was thus typed as *Protoparvovirus carnivoran1*. More studies, including serology, are needed to understand the pathogenesis of this infection.

## Simple Summary

One of the main threats to the Iberian lynx, an endangered species, is the possibility of infection by transmissible pathogens, including parvoviruses, a family of small and very resistant viruses. A lynx died with the signs of parvoviral infection. It developed the disease three weeks after being transported to a Recovery Centre from a hunting estate in the southern centre of the Iberian Peninsula, where a lynx population is known to inhabit, and died four days later. We screened the faecal material (*n* = 66) of other lynxes from the hunting estate using PCR but found no positive sample. However, we obtained the complete sequence of the parvovirus from the brain of the dead lynx. Taxonomically it has been typed as a feline parvovirus, in the species *Protoparvovirus carnivoran1*. To compare it with other circulating strains, we sequenced parvoviruses from a cat and a dog with parvoviral disease. The sequences of the lynx and the cat showed a high degree of similarity. They were also very closely related to other feline parvoviruses from Italy and Spain. This is the first description of the full genome of a parvovirus infecting the Iberian lynx associated with a clinical case, and points to the need to increase efforts to understand the pathogenicity of the disease process.

## 1. Introduction

The Iberian Lynx (*Lynx pardinus*) is one of the most threatened feline species worldwide. It is currently classified as “vulnerable” by the IUCN (International Union for Conservation of Nature) [1]. After 20 years of implementing various in situ and ex situ programmes, a population of 2021 individuals has been reached in the Iberian Peninsula in 2023, mainly located in the southwestern regions of Spain (Andalusia, Castilla-La-Mancha and Extremadura) [2].

The reasons for the dramatic situation that the Iberian lynx has reached are various, including a drastic decrease in the population of rabbits, the main food source for lynxes, collisions with vehicles and infectious diseases [3,4,5,6]. Between 2003 and 2011, infectious diseases such as pasteurellosis, tuberculosis, feline leukaemia virus and feline parvovirus infections, among others, were reported as the cause of 38.5% of the recorded deaths of lynxes in the Andalusia region [7]. More recent studies also reported the detection of feline leukaemia virus, feline coronavirus, feline calicivirus and feline parvovirus in the Iberian lynx in the Extremadura region [8].

It has been reported that the Iberian lynx has a lack of acquired and innate immunity and immunocompetence, maybe due to inbreeding [9]. Susceptibility is enhanced by sharing the habitat with other carnivore species, which may harbour pathogens but which may pass unnoticed in the management of the area [5,6,8]. Consequently, infectious diseases may pose a serious risk for lynx conservation [5,6]. As feline parvovirus represents a threat to the Iberian lynx [5,8], screening tests are routinely carried out.

Parvoviral infection, which may affect different species, is usually acute and frequently lethal. In cats, feline parvovirus (FPV) infection causes feline panleukopenia (FPL), a highly contagious disease characterised by acute severe enteritis, dehydration and sepsis due to lymphoid depletion and pancytopenia, which may be fatal [10]. Parvoviruses are transmitted by the faecal–oral route and are spread through contact with the faeces and body fluids from infected animals and fomites. The incubation time until clinical signs appear is usually two to ten days [11].

Both FPV and its close relative, canine parvovirus (CPV), belong to the genus *Protoparvovirus*, species *Protoparvovirus carnivoran1* within the family *Parvoviridae*, subfamily *Parvovirinae*. They are small, non-enveloped icosahedral viruses with a single-stranded DNA genome of approximately 5.2 Kb. FPV and CPV exhibit 98% genome homology [12,13]. The coding region of the genome contains two major open reading frames (ORFs): the 5′ ORF (ORF1) gives rise to non-structural (NS1 and NS2) proteins, whereas ORF2 translates into structural proteins (VP1 and VP2) [12]. NS proteins are essential for replication and DNA packaging, and VPs form the capsid and determine viral entry processes and host immunity [14]. The ends of the genome usually consist of untranslated regions (UTR) that form hairpin structures necessary for the initiation of replication [15,16].

Parvoviruses lack the ability to induce cell multiplication and for the replication process to occur, they must infect cells that are in the S-phase of division, thus exhibiting tropism for tissues with high mitotic activity, such as the epithelial cells of the intestinal crypts, bone marrow and lymphoid tissues, and foetal cells [17,18]. Viral replication begins in the lymphoid tissue associated with the oropharynx, followed by a viraemia lasting 2–7 days, during which it is distributed to other lymphoid organs such as the spleen or bone marrow, producing leukopenia and transient immunosuppression. It finally spreads to virtually all tissues, and in pregnant females, the virus crosses the placenta and infects the foetus. The ensuing shortening of the intestinal villi causes a malabsorption syndrome that triggers diarrhoea, which may be haemorrhagic or non-haemorrhagic, along with other associated clinical signs such as anorexia, dehydration, vomiting, and generalised weakness. This clinical outcome makes parvoviral infection one of the most severe diseases in cats. Depending on the viral strain, immune status and age of the affected animal, FPV may produce its death [19,20]. Infection by FPV can be also subclinical, mainly in adult and subadult cats, which shed virus in their faeces. This is supported by the high seroprevalence rates in some populations of unvaccinated adult cats [10], and by the probable persistence of the virus in the tissues of animals that have recovered from the infection [21].

Veterinary practitioners usually suspect a disease based on the clinical signs, but confirmation by laboratory tests is recommended, of which PCR, both end-point or conventional (PCR) and quantitative or real-time (qPCR), is considered definitive.

Parvoviruses have a large host spectrum [19]. Infection by FPV occurs worldwide and all members of the family Felidae are probably susceptible to infection with this virus [19]. Other carnivores of the families Viverridae, Procyonidae, Canidae and Mustelidae have been reported to also be susceptible to infection with these viruses [19]. Cases of parvovirus infection and/or disease have been described in lion, Eurasian lynx, tiger, leopard, cheetah and panther, besides Iberian lynx [4,17,22,23,24]. Clinical disease has been observed in raccoon (*Procyon lotor*), mink (*genera Mustela* and *Neovison*), coatimundi (*genus Nasua*) [25] and leopard (*Panthera pardus*) [26]. Wild and domestic carnivore species sharing territory with the lynxes can act as reservoirs of FPV, favouring its transmission [5,20,22,27,28,29]. Pathogenesis and disease outcomes of this viral disease have been mainly studied in domestic cats, and the infectivity of parvoviruses in the Iberian lynx is mostly unknown, though both the virus (a sign of active infection) [3,8,27] and antibodies (a sign of past or present exposure to the virus) [5,6,8] have been detected in this threatened species.

In January 2023 in Castilla-La-Mancha (central Spain), three weeks following translocation from a hunting estate for repopulation reasons, a subadult lynx was observed to have poor body condition, anorexia, diarrhoea and muscle weakness. Consequently, a veterinary examination was performed at the wildlife recovery centre “El Chaparrillo” in Castilla-La-Mancha. Despite stabilization attempts, the animal died four days later. A commercial qPCR for feline parvovirus diagnosis was performed with a positive result. The aim of the present study was to describe, for the first time, the complete sequence of a parvovirus genome from an Iberian lynx from the southern part of the Spanish plateau, which may be associated with its cause of death. As the possibility existed that it might have been infected from sympatric domestic animals, and to determine if this sequence was related to other circulating parvoviruses in Spain, the parvoviral sequence of a cat and a dog from central Spain (though not exactly from the same area as the lynx) were also sequenced. All three of the sequences were assigned to the species *Protoparvovirus carnivoran1*. According to the VP2 amino acid sequence, the detected parvovirus from the lynx was typed as feline parvovirus.

## 2. Materials and Methods

### 2.1. Case Report

**Figure 1:**
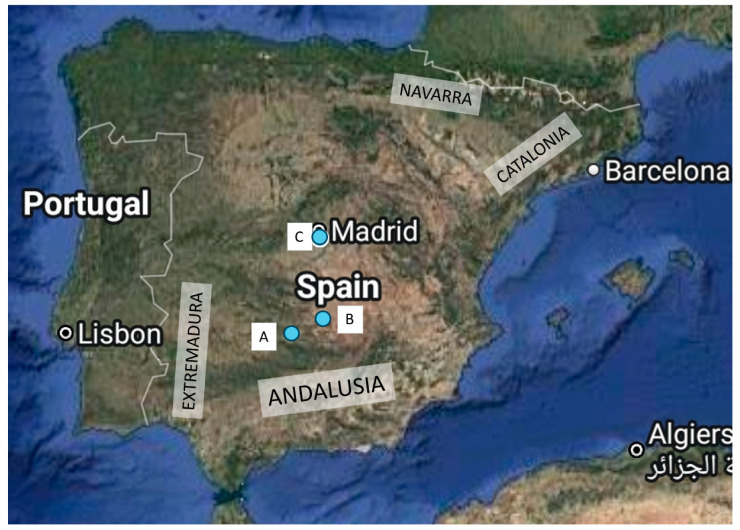
Geographic position of the hunting estate (A), Recovery Centre “El Chaparrillo”, Ciudad Real (B) and Madrid (C) (http://google.com/maps). The regions of Andalusia, Extremadura, Catalonia and Navarra, mentioned in the text, are also shown.

A subadult (as judged by the dentition) male Iberian lynx was captured in a hunting estate located in Ciudad Real (a province in the region Castilla-La-Mancha, in the southern limit of the Spanish plateau) for translocation and repopulation reasons in December 2022 in Castilla-La-Mancha (Figure 1). The lynx population in this hunting estate is estimated to be between 150 and 200 individuals. It is fenced all around, but lynxes have been observed to jump the fence in both directions. The lynx was transferred to the Recovery Centre “El Chaparrillo” in Ciudad Real, 93 km from the capture area, for quarantine. Upon arrival it was subjected to routine testing with no evident alterations in any of the parameters and no visible signs of illness. Feline leukaemia virus (FeLV) and feline immunodeficiency virus (FIV) rapid tests were negative. A rectal swab was sent to an external laboratory (Centre for the Analyses and Diagnosis of Wildlife in Andalusia, CAD, the reference laboratory for the Iberian lynx) for qPCR detection of FPV, which gave a borderline result (Ct 33). The lynx was kept in a species-appropriate quarantine facility with a 9 m^2^ box and a 400 m^2^ outdoor area for three weeks, after which it developed acute disease, including severe diarrhoea, anorexia and dehydration. Despite stabilization attempts (fluid administration, antibiotic therapy and analgesia), it died four days later, and a complete necropsy was performed. Samples were taken for histopathological analysis, performed by an external company (Urano Diagnostics), where they were processed following standard procedures. A rectal swab obtained when the lynx was sick and a sample of a mesenteric lymph node taken at necropsy were analysed by qPCR by the same external laboratory, giving positive results of Ct 17.93 and 20.18, respectively.

### 2.2. Samples

Sampling was carried out between March 2023 and March 2024 in the same hunting estate as described above. The sample set included *n* = 51 stool samples; *n* = 15 rectal swabs and *n* = 15 serum samples from live Iberian lynxes; and *n* = 12 stool samples from dogs, which were clinically healthy, from the same hunting estate as the lynxes. The 15 Iberian lynxes were captured for a sanitary survey and released after. All samples obtained from them were extracted under strict compliance of the protocols established for the health monitoring of the species by the Iberian Lynx Health Advisory Group (GAAS) [30]. To determine the similarities with other parvoviral strains circulating in Spain, stools from a diarrhoeic cat (LG15) and dog (LG151), both from Madrid, were also analysed. They had been confirmed positive for parvovirus by an in-practice immunochromatography test and were used as positive controls. The pet owners had been informed about this study and had agreed to participate in it. Faecal samples were suspended in RNAlater (Thermo Fisher, Boston, MA, USA).

In addition, we analysed samples of the brain and heart, obtained from the necropsy of the dead lynx mentioned above, following a standard necropsy procedure. All samples were stored at −20 °C until DNA isolation was carried out.

### 2.3. DNA Extraction

Rectal swabs, faeces and necropsy tissues were resuspended in 1 mL of phosphate-buffered saline (PBS). After mechanical maceration, samples were centrifuged at 4000 rpm for 2 min; this facilitated the proper dilution of the sample in the added PBS and the sedimentation of solid matter at the bottom of the Eppendorf tubes, preventing obstruction of the extraction kit membrane. DNA was extracted from the supernatant using the High Pure Viral Nucleic Acid Kit (Roche, Mannheim, Germany) following the manufacturer’s instructions and stored at −20 °C.

### 2.4. Commercial End-Point PCR

All DNA extracts from the lynx, cat and dog samples were analysed by a commercial end-point PCR for canine and feline parvovirus (CPV-2-FLPV-EPPCR, Genetic PCR solutions™, Orihuela, Alicante, Spain), following the instructions of the manufacturer. Amplified products were visualised in 0.8% agarose gels. A band of the expected size (around 300 bp) in the electrophoresis gel was considered positive.

### 2.5. In-House Nested PCR

To confirm the results, all DNA extracts from the lynx, cat and dog samples (faeces, rectal swabs and necropsy samples) were further screened by a nested PCR, designed by us, targeting the NS1 region of the parvoviral genome using an external PCR, which amplified a 1671 nucleotide (nt) segment using the primers FPL28Fw and FPL1699Rev (Supplementary Table S1), followed by an internal PCR, giving a product of expected size of 959 nt, using the primers FPL438Fw and FPL1397Rev (Supplementary Table S1) at a 1:10 dilution of the first PCR product. Primers were designed by comparing 21 parvoviral genomes deposited in GenBank (Supplementary Table S2). This comparison included sequences from cats, dogs and wild animals from different areas of the world which were aligned and analysed using MEGA software version 11 (MEGA11), selecting the most conserved areas. The primers were analysed using Vector NTI software (Thermo Fisher Scientific, https://www.thermofisher.com/ro/en/home/life-science/cloning/vector-nti-software.html, accessed on 27 March 2025). The DNA extracted from the cat LG15 was used as a positive control, and the non-template reaction was included as a negative control.

**Figure 2:**
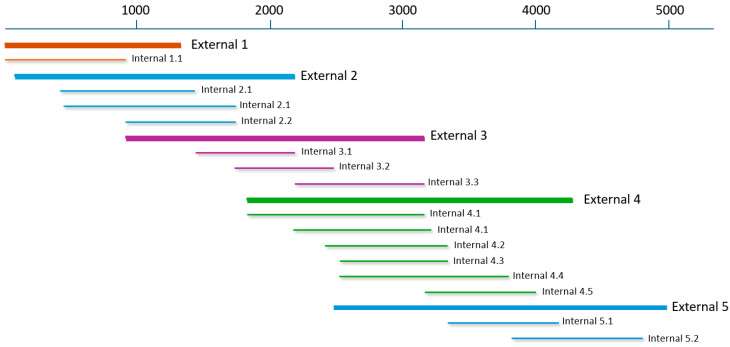
Strategy of nested PCRs used for sequencing the complete genome of *Protoparvovirus carnivoran1* from lynx (LG100), cat (LG15) and dog (LG151). The thin line on top represents the parvoviral genome. The thick lines represent external PCRs, while the thinner ones with shadow represent internal PCRs. The exact position of each primer is shown in Supplementary Table S2.

Samples of the brain from the lynx, and the faeces from a cat and dog were subjected to complete genome amplification using a total of 17 sets of primers designed by us (Supplementary Table S1), following a similar strategy as described above. The most conserved regions of 18–23 nt, separated 500–1000 nt from each other, were used for the design of primers covering the entire parvovirus genome (detailed in Supplementary Tables S1 and S3, and Figure 2). Nested PCRs included external PCRs, which could be up to 2500 nt in size, and internal PCRs, which were 900–1100 nt in size (Supplementary Table S3). For sequencing the dog parvovirus, primers published by Pérez et al. [15] were used to complete the difficult-to-sequence areas.

Nested PCRs were performed using DNA Polymerase (1 U/µL; Biotools, Madrid, Spain) in a final volume of 25 µL comprising 16 or 14 μL of sterile distilled water (external or internal PCR, respectively), 2.5 μL of Buffer 10× with MgCl_2_ (Biotools), 0.5 μL dNTPs (10 mM Biotools), 1 μL of each primer (10 μM), 1 μL DNA Polymerase (1 U/µL) and 3 or 5 μL of DNA (external or internal PCR, respectively). The first PCR product was diluted in a 1:10 ratio and used as template for the internal PCR. Extreme care was taken to avoid PCR cross-contamination, and the DNA extraction, external PCR, internal PCR and separation in the gel were handled in separate work areas. Non-template controls were included in each assay to ensure that there was no sample contamination, which was also confirmed by the consistency of the results. The PCR amplification was performed with the following cycle conditions: initial denaturation at 95 °C for 5 min; followed by 30 cycles of denaturation at 95 °C for 1 min, annealing at 5 °C below the lowest Tm of the pair of primers for 1 min, extension at 72 °C for 2 or 1 min (external or internal PCRs, respectively, Supplementary Table S3); and final extension at 72 °C for 5 min.

### 2.6. Sequencing and Further Analyses

The amplified PCR products were purified using the QIAquick PCR Purification Kit (Qiagen, Hilden, Germany) and Sanger’s dideoxy-based sequencing was performed using both forward and reverse primers at Macrogen, Inc. (Seoul, Republic of Korea). The nucleotide sequence data were checked with Chromas Version 2.6.6 (Technelysium DNA Sequencing Software), subjected to Blast analysis, and assembled and analysed using MEGA11 by the ClustalW method. The amino acid sequence, predicted using ORF Finder (https://www.bioinformatics.org/sms2/orf_find.html, accessed on 14 May 2024), was also subjected to Blast search and analysed with MEGA11. Assembled sequences were deposited in GenBank with the accession numbers PP781551 (lynx parvovirus LG100), PQ436979 (feline parvovirus LG15), and PQ436980 (canine parvovirus LG151).

Phylogenetic analysis of the obtained genomes was performed using the Clustal Omega core alignment engine (https://www.ebi.ac.uk/jdispatcher/msa/clustalo (accessed 27 March 2025)), which uses the HHalign algorithm, and its default settings. The algorithm is described in [31]. The GenBank accession numbers of the 51 sequences of *Protoparvovirus* included for constructing the phylogenetic tree of the VP2 protein are shown in Supplementary Table S4. These were selected to cover a large spectrum of mammalian species hosts from different parts of the world.

### 2.7. Serological Analysis

Sera of the 15 Iberian lynxes captured for sanitary check-ups were analysed by ELISA for the detection of antibodies against FPV (IgG and IgM) (Feline Parvovirus ab EIA, Eurovet Veterinaria, Daganzo, Madrid, Spain), following the manufacturer’s instructions.

## 3. Results

### 3.1. Necropsy Findings of the Affected Iberian Lynx

**Figure 3:**
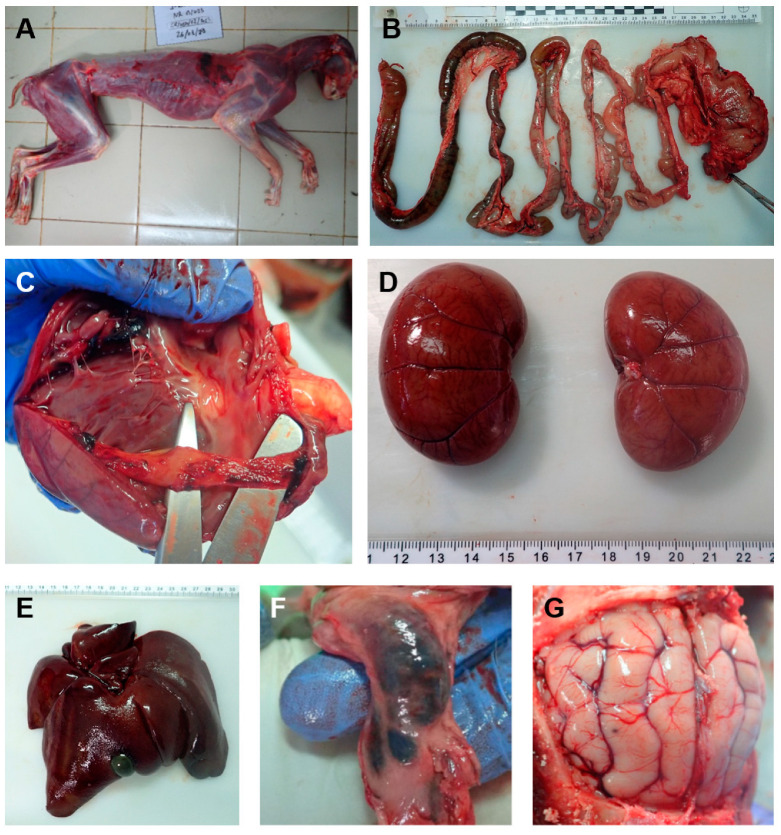
Necropsy findings of the affected Iberian lynx. (**A**) The body condition of the lynx was judged as 1.5 on a scale of 1–5. (**B**) Congestion of the submucosal vessels and haemorrhagic enteritis. (**C**) Presence of congestion in the heart. (**D**) Kidney congestion. (**E**) Hepatomegaly and hepatic congestion. (**F**) Congestion and enlargement of the mesenteric lymph node. (**G**) Congestion of the encephalic vessels.

Upon death, the affected lynx was found to be severely emaciated, with a body condition of 1.5 (scale 1–5) (Figure 3A). The necropsy (Figure 3) revealed the presence of haemorrhagic gastroenteritis with the congestion of several organs, including the subcutaneous and submucosal vessels, mesenteric lymph nodes, ileocecal papilla, brain vessels and spleen, liver congestion and hepatomegaly, lung congestion, and oedema and emphysema, among other lesions, which were all compatible with parvovirus infection or secondary bacterial infections due to increased intestinal permeability caused by the virus. Cardiomegaly and myocarditis were also observed but were not confirmed by histopathology. The wall of the bladder was engrossed.

The results of the histopathological study revealed encephalitis with multifocal satellitosis, centrilobular atrophy and hepatic congestion, multifocal interstitial nephritis, congestion and pulmonary oedema with diffuse interstitial pneumonia, and a reactive lymphoid population in the mesenteric lymph node. The sent intestinal fragments presented a high degree of autolysis, which hampered the correct assessment of the mucosa.

Samples from both the brain (LG100) and the heart (LG101) obtained in the necropsy of this lynx yielded a positive result for parvovirus by conventional PCR, both through commercial methods and in-house methods designed by us targeting the NS1 gene with 982 bp product (external primers FPL28Fw/FPL1699Rev; internal primers FPL438Fw/FPL1397Rev). DNA extracted from the brain was subjected to complete genome sequencing and was submitted to GenBank where it received the accession number PP781551 (LG100lynx). Several PCR reactions with other primers were also performed with the other positive sample from the same lynx (LG101), and as the sequencing results were identical to LG100, it was not sequenced any further. This sequence shared 99.76% nucleotide identity (based on the complete genome sequence) with the sequence of a feline panleukopenia virus strain (accession number OP588004), isolated from a Eurasian badger (*Meles meles*) in Italy, the closest relative so far in GenBank. The genomic characteristics of the LG100lynx sequence are shown in Table 1. According to the VP2 amino acid sequence, the LG100lynx sequence was typed as feline parvovirus.

### 3.2. Prevalence of Parvovirus in the Hunting Estate

To determine the prevalence of the infection in the hunting estate, we screened, by both a commercial PCR and with our in-house nested PCR, DNA extracted from the faeces of local lynxes. Rectal swabs from the 15 individuals captured for sanitary survey reasons were also analysed. None of these 66 faeces and rectal swab samples were found to be positive. To determine the origin of the infection of the affected lynx, the faeces of 12 local dogs were also analysed, which all tested negative.

In addition, all sera from the 15 live lynxes captured for sanitary screening, were negative by ELISA to antibodies against feline parvovirus.

### 3.3. Parvoviral Sequences from a Cat and Dog in Spain

In an attempt to determine the origin of the infection in the lynx by comparison with the sequences of parvoviruses from other animals and in the absence of positive samples in other species in the hunting estate, stools from a parvovirus-infected symptomatic cat (LG15) and dog (LG151) from Madrid were analysed. Both tested positive by the commercial and in-house conventional PCRs. They were also subjected to complete genome sequencing, and the sequence data were deposited in GenBank with accession numbers PQ436979 (LG15cat) and PQ436980 (LG151dog), respectively. LG15cat shared 99.87% nucleotide identity with the OP588004 FPV variant, isolated from the Eurasian badger, and 99.82% nucleotide identity with the OR602718 and OM638042 FPV variants, isolated from a crested porcupine and a dog, respectively. All three of them were reported by Italian researchers. LG151dog shared 99.93% nucleotide identity with the MG013488 and MH476583 CPV variants, isolated from dogs in China. LG100lynx shared 99.87% nucleotide identity with LG15cat and 98.52% with LG151dog. The genomic data of LG15cat and LG151dog are shown in Table 1.

According to the deduced amino acid sequence of VP2, LG15cat was typed as FPV, while LG151dog was typed as CPV-2c. The comparison of the deduced amino acid sequences of VP2 from the strains isolated in this study with other FPV and CPV-2 strains from different countries is shown in Supplementary Table S4. The VP2 sequence of two parvoviral descriptions from Spain (from a stone marten (KP682526) and an Eurasian badger (KP682520 [20]), three from Italy (a dog (OM638042), a cat (KX434461) and a Eurasian badger (OP588004)), and two from China (from a cat (MG924893) and a jaguar (KX900570)) were exactly like LG100lynx. Likewise, the VP2 sequence of the parvovirus of six dogs in China (MG013488, MT648203, MH476583, MW650830, MF805796, MN519258), one from a dog in Vietnam (MT106233), one from a dog in Italy (OP588002), and one from a Pangolin in Taiwan (MN832850)) were exactly like the VP2 sequence of LG151dog.

### 3.4. Phylogenetic Analysis of VP2

**Figure 4:**
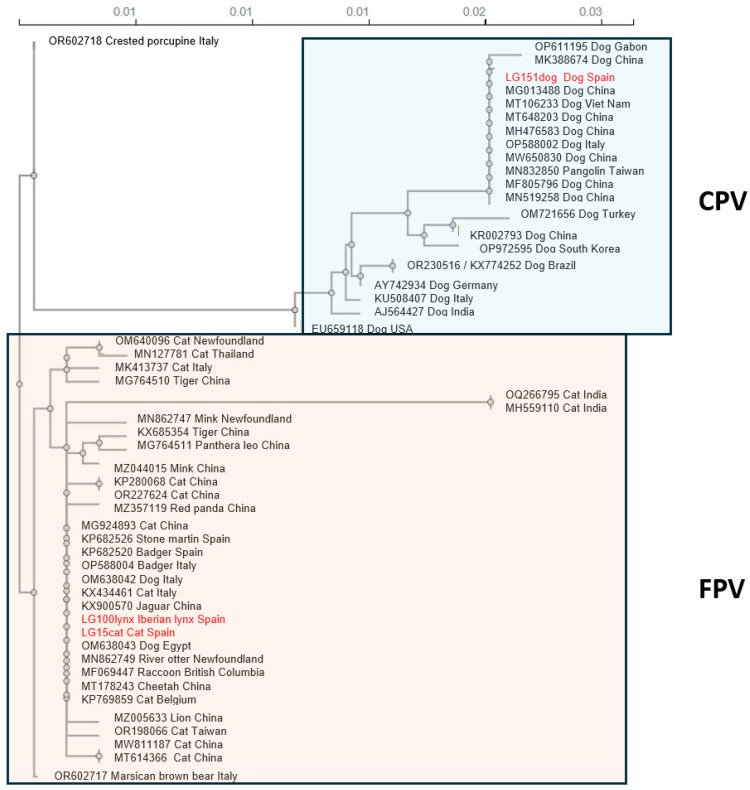
Phylogenetic tree based on the amino acid sequences of the full-length VP2 protein from Spanish strains sequenced in this study (indicated in red) and other strains (shown by GenBank accession number, host species and country of isolation). Blue box, strains typed as CPV. Pink box, strains typed as FPV.

A phylogenetic tree based on the protein encoded by the full-length VP2 gene was constructed using fifty-one FPV and CPV-2 sequences, forty-eight of them obtained from GenBank and three isolated in this study (LG100lynx, LG15cat, LG151dog). (Figure 4, Supplementary Table S5). All of the obtained sequences were from sequences listed as complete genome in GenBank.

## 4. Discussion

The Iberian lynx has just passed from the IUCN list of “critically endangered species” to that of “vulnerable species” as a result of conservation efforts. The population has significantly increased thanks to captive breeding projects and reintroduction. While the population was initially 62 mature individuals in 2001, it has increased to 648 in 2022. Today, the total population, including young and mature ones, is estimated to exceed 2000 individuals [1]. However, the situation is far from ideal, as lynxes are exposed to a multitude of dangers, infectious diseases being one of the most prominent threats. In this context it is especially important that lynxes which are being translocated to repopulate other areas are absolutely free of known pathogens. The lynx in this report was found to have a borderline result with a commercial qPCR test for feline parvovirus (Ct 33) in a rectal swab at its entrance to the Recovery Centre “El Chaparrillo”, where it had been taken for quarantine as an intermediate stage in the translocation process. Almost four weeks later, it died from acute disease and qPCR results showed an evident increase in the parvoviral load (Ct 17.93 in rectal swab), confirming for the first time the presence of the virus in this Spanish region. Several questions were raised, including whether other lynxes were infected, where the infection came from, and whether the lynx was already infected before its capture in the hunting estate.

To determine if other lynxes were infected, faeces were screened to ascertain the presence of parvovirus in these samples. Stool samples were chosen as a non-invasive and adequate way to determine parvoviral infection in carnivores [3]. When analysing the results, it must be taken into account that two or more faecal samples could proceed from the same animal so the number of stool samples may be higher than that of the lynxes scrutinised. During the autumn health monitoring campaign (10 months after the detection of the positive case), rectal swabs and blood samples from 15 Iberian lynxes, belonging to the same subpopulation as the dead lynx and captured for a sanitary survey, were also analysed. All stool samples and rectal swabs turned out to be negative for FPV (0/66), as well as for antibodies in the sera from the same animals as the rectal swabs (0/15), suggesting that the prevalence of the parvoviral infection in the hunting estate could be null or minimal.

This low prevalence of parvovirus in the Iberian lynx in the southern part of the Spanish central plateau is consistent with other studies reporting low exposure to this pathogen in this species. Najera et al. [8] detected a prevalence of 1.5% (1 lynx out of 67 sampled); the infected lynx died because of vehicle collision and no signs associated to FPV were observed at necropsy. López and collaborators analysed twenty-two fresh carcasses of Iberian lynxes in southern Spain (Andalusia) and identified only one in which death could be due to parvoviral infection [7]. This agreed with data from Meli et al. (2009), who studied different pathogens in two areas of Andalusia and found 2/75 (2.7%) free-ranging lynxes positive by PCR for FPV, both of them distant from the site of our study. A similar low prevalence has been observed in other lynx species worldwide [3,4]. The solitary and territorial nature of lynxes may account for this low prevalence, as contagion between individuals would be more difficult. However, serological surveys have detected the presence of anti-FPV antibodies in a range from ≤11% of non-vaccinated Iberian lynxes [6,8] and Eurasian lynxes [32] to 30% [33]. In a serological survey performed in 2023 in Castilla-La-Mancha, three out of sixty-four (4.7%, co-author Elena Crespo, personal communication) of the lynxes screened tested positive to parvoviral antibodies, confirming a previous but infrequent contact with the virus. In the present study and due to its non-invasive design, serology was performed only in the lynxes from which the rectal swabs were obtained. As mentioned, all of them tested negative. However, due to the limited sample size, more studies into serology would be advisable, especially taking into account that in lynxes, the infection may be subclinical, as highlighted by the finding of parvoviruses in the carcass and vehicle runovers mentioned above [7,8], and this would be fundamental for understanding the pathogenesis of this infection in the Iberian lynx.

To trace the potential origin of the infection in the lynx, we resorted to sequencing the genome of the virus. According to the genomic organization and phylogenetic inference of LG100lynx, the detected virus belongs to the *Protoparvovirus carnivoran1*. To the best of our knowledge, this is the first time that the complete sequence of a *Protoparvovirus* genome has been described in the Iberian lynx (*Lynx pardinus*), and that it is the first associated with a clinical case in the Castilla-La-Mancha region in Spain, as previous findings corresponded to dead animals and that the presence of parvovirus could not be associated with the death of the lynxes (they were found in a decomposed carcass and in runovers) [7,8].

The 12 dogs sampled from this location turned out to be negative. In the absence of finding parvoviruses in the hunting estate, we sequenced an FPV case and a CPV case from the central area of the country. LG100lynx had a high similarity to LG15cat (99.87%). Blast analysis showed that both LG100lynx and LG15cat were very closely related to other sequences previously found in different domestic carnivores from distant countries. This further supports that cross-species transmission takes place [20]. However, our results did not allow us to ascertain where and from which species the Iberian lynx may have obtained the parvoviral infection. In Spain, multiple parvoviruses have been found in different species of wild carnivores, including European wildcats, Eurasian badgers, a genet, and wolves (CPV-2c); wolves, red foxes and stone martens (CPV-2b); and badgers, genets and stone martens (FPV) [20,27]. Studies about the presence of FPV in areas where there is a population of Iberian lynxes suggest FPV circulation and that it may have a reservoir in free-roaming domestic cats [5]. The well-known environmental resistance of parvoviruses would facilitate transmission.

To further compare LG100lynx with other parvoviruses, the amino acid sequence of VP2 was characterised. This is the most abundant protein of the capsid in parvoviruses [34] and determines the host range and antigenicity, and variations in the VP2 gene are closely associated with the virulence and transmissibility of the virus. It is crucial for typing strains into CPV and FPV [20], and specific amino acid positions differentiate the CPV-2a, -2b and -2c variants [35,36]. Within FPV, it is worth mentioning that, as seen in the phylogenetic tree (Figure 4), isolates from one dog in Italy (OM638042), and one dog from Egypt (OM638043) fell outside of this species; and that the Spanish sequences were most similar to other strains from Spain and Italy. The similarity in the sequence we obtained from the symptomatic cat in central Spain (LG15), and with the VP2 sequences of the stone marten (*Martes foina*) and two Eurasian badgers, one obtained in Catalonia, in north eastern Spain [20], and a second one in Navarra, in northern Spain [37], suggests LG100lynx might be derived from currently circulating FPVs in the Spanish cat population, as observed in other studies, in which the possible transmission of FPV from domestic to wild animals has been described [4,5,28,29,37]. Further research is needed to understand the dynamics of FPV transmission between the Spanish wild and domestic environments in this particular case, as well as the origin of the infection in the Iberian lynx. As expected, LG151dog exhibited similar amino acid variations as the other sequences typed as CPV-2c [20]. Interestingly, the LG151dog sequence is closely related to a CPV-2c strain recently detected in Spanish wild carnivorans, belonging to the so-called new “Asian CPV-2c” lineage, which is spreading rapidly worldwide, but to date has not been described in domestic dogs in Spain [37]. Although it is not the objective of this paper, our description of a possible “Asian CPV-2c” lineage in a dog from the central region of Spain could be the first evidence that this virus is already circulating among Spanish pets in addition to carnivoran wildlife, according to the proposal by Canuti et al. [37]. This is in an unexpected but very interesting result that requires further studies.

As to whether the lynx had been infected before it was quarantined, evidence points to a subclinical infection, as qPCR at entrance in the Recovery Centre was borderline though present (Ct 33). The onset of the infection could be attributed to captive and transport stress. Whether certain conditions such as immunosuppression could induce the re-shedding of FPV or CPV in cats had not been investigated (in [10]), but other similar cases have been reported in wild animals [26]. It has been suggested that *Protoparvovirus carnivoran1* DNA may persist for long periods in the tissues of animals that have recovered from infection without an active infection [21]. Subclinical infections, common in young adult cats, are difficult to detect, and may become apparent only when the animal is subjected to stress [10].

The fact that both the brain and the heart of the affected lynx were positive to parvovirus by PCR is intriguing. These are sites that may be affected in foetal kittens but not in adult cats, though it has been reported that parvoviruses may replicate in the cerebral neurons of cats [38]. A specific mutation in the protein NS1, L582S, that may be related to the neuronal tropism of FPV [39] even in adult cats, was not detected in the LG100lynx sequence, which coincides with that described in other FPV strain recovered from the central nervous system in cats [40]. Thus, it is difficult to conclude that this mutation may be involved in the neural tropism of FPV and more studies are necessary to characterise the pathogenesis of FPV strains isolated from brain tissue.

In our study, the congestion of encephalic vessels was evident, and the positive detection of parvovirus by PCR and its subsequent typing as FPV constitutes the first report of infection by this virus in the brain of an Iberian lynx. This data may be relevant for the epidemiology of FPV in lynx, as the animal, a subadult, could be suffering an asymptomatic infection with the presence of the virus in brain areas without neurological signs, since anorexia and diarrhoea were the only clinical manifestations observed. It is worth mentioning that pathogenesis studies stem from data in cats and the progress might be different in other Felidae. The absence of the specific mutation in NS1 [39] also broadens the spectrum of strains that have tropism for brain tissues. With regards to myocarditis, it is a recognised complication of CPV infection in puppies, but it has not been convincingly associated with FPV in cats [10]. Unluckily, the other tissues were not made available to us and would have helped to clarify the extension of the infection in this lynx, though histopathological analysis provided data as to the pathogenesis of the infection.

The increase in the viral load of FPV, as judged by the increase in Ct by qPCR, suggested an association of the process with feline parvovirus. However, the presence of anorexia, vomiting, diarrhoea, dehydration and weight loss in the Iberian lynx could have also corresponded to progressive infection by feline leukaemia virus (FeLV), alone or in coinfection with FPV. This is referred to as feline panleukopenia-like syndrome (FPLS), and has been described in cats [41] and, more recently, in a Eurasian lynx (*Lynx lynx*) in Germany [42]. This hypothesis was discarded as the FeLV test was negative. Finally, other causes such as a foreign body or a severe gastrointestinal parasitism were discarded at necropsy. Although most cases of mortality in FPV infection are due to a secondary bacterial infection associated with the invasion of intestinal bacteria through gastrointestinal epithelium destroyed by the virus [10,18,43], this could not be analysed in our samples.

## 5. Conclusions

In conclusion, the clinical signs, lesions and histopathological findings were consistent with parvoviral enteritis, which was confirmed by increased viral shedding in the faeces of the clinically ill lynx (Ct 17.93) compared to the beginning of the quarantine period when the lynx was asymptomatic (Ct 33), indicating an active and acute infection. In addition, post-mortem FPV was detected in the mesenteric lymph node (Ct 20.8) and brain (by commercial and in-house conventional PCR) of the Iberian lynx. All these results strongly suggest that the animal suffered an acute process probably associated with FPV infection. Although FPV infection or the exposure to the virus has been previously reported in Iberian lynx by other authors, to the best of our knowledge, the clinical disease has never been documented in this species [6]. Based on the healthy condition of the lynx at the time of admission and the time it took to develop the disease, our hypothesis regarding the described case is that it had a subclinical FPV infection which become apparent when it was subjected to stress. More studies, including serology to determine previous exposures to the virus, and body locations, where the virus may persist due to its resistance, are needed to determine the pathogenicity of FPV in the Iberian lynx and to prevent its spread, endangering the survival of the species even further.

Infectious diseases can be a threat to the survival of endangered species, so the healthcare of populations is of the utmost importance. Specifically in the Iberian lynx, the lack of immunocompetence associated with consanguinity derived from the limited number of animals makes it especially vulnerable. On the other hand, the comeback of this species implies the occupation of suboptimal territories, closer to urban centres that involve a greater probability of contact with domestic cats. The naivety of the lynx immune system to parvoviruses due to little contact with these pathogens, as shown by serological analyses, makes these viruses a potential threat to the populations of Iberian lynx. Since this virus may cause high mortality in the Iberian lynx [7], periodic screening should be conducted in these animals to prevent and monitor possible cross-transmission from domestic cats or other wild species.

## Figures and Tables

**Table 1:** Genomic characteristics of the three sequences obtained in this study.

GenBank Acc. No.	Host	Sample	Total nt Sequenced	ORF1 NS1 and NS2	ORF2 VP1 and VP2
PP781551	*Lynx pardinus*	LG100	4589	nt 64–2070	nt 2077–4333
PQ436979	*Felis catus*	LG15	4543	nt 52–2058	nt 2065–4320
PQ436980	*Canis lupus domesticus*	LG151	4478	nt 23–2029	nt 2036–4291

## Data Availability

Parvoviral sequences obtained from this study have been deposited in GenBank under accession numbers PP781551 (Iberian lynx LG100), PQ436979 (cat LG15) and PQ436980 (dog LG151).

## Supplementary Materials

The following supporting information can be downloaded at: https://www.mdpi.com/article/10.3390/ani15071026/s1, Figure S1. Phylogenetic tree of the 32 genome sequences deposited in GenBank with which the LG100lynx, LG15cat, and LG151dog (marked with red stars) sequences were compared, including the species from which they were isolated and the geographic location; Table S1. Primers used in the study. Nucleotide positions in the genome of *Protoparvovirus carnivoran1* sequenced from a badger in Italy (GenBank Acc. No. OP588004). Primers were named according to the position of the first nucleotide in the + strand. UTR, untranslated region. Fw, forward primer. Rev, reverse primer. All primers were designed by us, except for those marked with **, which were published before [15]. Table S2. GenBank accession numbers of the 21 parvoviral genomes used for designing the primers used in this study, including the species in which they have been typed by their researchers. Table S3. Protocols of the nested PCRs used for sequencing the complete genome of *Protoparvovirus carnivoran1* from lynx (LG100), cat (LG15) and dog (LG151). The number in the name of the primer, where present, indicates the position in the genome of *Protoparvovirus carnivoran1* sequenced from a badger in Italy (GenBank Acc. No. OP588004). Table S4. Amino acid comparison of the VP2 region between the strains from this study with other FPV and CPV-2 strains from NCBI. Sequences obtained in this study are shown in bold. VP2 amino acid sequences of stone marten (KP682526), Eurasian badger (KP682520) isolated in Spain [20], dog (OM638042), cat (KX434461) and Eurasian badger (OP588004) isolated in Italy, cat (MG924893) and jaguar (KX900570) isolated in China, were exactly like LG100lynx and are not included in the table. Likewise, the VP2 amino acid sequences of dogs (MG013488, MT648203, MH476583, MW650830, MF805796, MN519258) isolated in China, Vietnam (MT106233) and Italy (OP588002), and in a pangolin (MN832850) from Taiwan were exactly like LG151dog and are not included in the table. Table S5. GenBank accession numbers of the sequences used for constructing the phylogenetic tree of the VP2 protein, including the species in which they were typed by their researchers. Only sequences listed in GenBank as complete genomes were used for this construction.
