# Supplementary material for: First Description of a Carnivore Protoparvovirus Associated with a Clinical Case in the Iberian Lynx (Lynx pardinus)
Source: Animals (Basel). 2025 Apr 2;15(7):1026. doi: 10.3390/ani15071026 (PMC11988045; doi:10.3390/ani15071026)
Supplement: Supplementary file 1 [file animals-15-01026-s001.zip › animals-3538774-supplementary.pdf]

Supplementary Table S1. Primers used in the study. Nucleotide positions in the genome of *Protoparvovirus carnivoran 1* sequenced from a badger in Italy (GenBank Acc. No. OP588004). Primers were named according to the position of the first nucleotide in the + strand. UTR, untranslated region. Fw, forward primer. Rev, reverse primer. All primers were designed by us, except those marked with **, which were published before [16].

Primer	Sense	Nucleotide positions	Sequence 5'→3'	Tm (°C)
FPLutr1Fw	+	UTR	CCA ACT GAC CAA GTT CAC GTA C	63.0
FPLutr2Fw	+	UTR2	GTG GGC GTG GTT AAA GG	60.1
FPLutrgato1Fw*	+	UTR	GTT CAC GTA CGT ATG ACG	57.3
FPLutrgato2Fw*	+	UTR2	GYT ACT GRC ATT CGC TTC TTG	61.4
FPL28Fw	+	28 - 48	GTT ATG GAG GGA GTA AAT TGG	58.2
FPL438Fw	+	438 - 459	GCT ACG CAG ACA AAT GAA TAT G	59.9
FPL438Rev	-	438 - 459	CAT ATT CAT TTG TCT GCG TAG C	59.9
FPL937Fw	+	937 - 955	GAA ATG ATG GCA CAA CCA G	59.1
FPL937Rev	-	937 - 955	CTG GTT GTG CCA TCA TTT C	59.1
FPL1397Fw	+	1397 - 1418	GAA TTG ATC AAA AAG GTA AAG G	56.4
FPL1397Rev	-	1397 - 1418	CCT TTA CCT TTT TGA TCA ATT C	56.4
FPL1699Fw	+	1699 - 1718	GAG CCT AAA ATA CAA GAA GG	55.9
FPL1699Rev	-	1699 - 1718	CCT TCT TGT ATT TTA GGC TC	55.9
FPL2158Fw	+	2158 - 2176	AGT CTT GAC CAA GGA GAA C	58.8
FPL2158Rev	-	2158 - 2176	GTT CTC CTT GGT CAA GAC T	58.8
FPL2384Fw	+	2384 - 2659	GCA ATT GCT CCA GTA TTA ACT G	59.9
FPL2384Rev	-	2384 - 2659	CAG TTA ATA CTG GAG CAA TTG C	59.9
FPL2509Fw	+	2509 - 2525	GCA CCA ATG AGT GAT GG	57.5
FPL2509Rev	-	2509 - 2525	CCA TCA CTC ATT GGT GC	57.5
FPL3109Fw	+	3109 - 3127	CCA TGG AAA CCA ACC ATA C	58.3
FPL3109Rev	-	3109 - 3127	GTA TGG TTG GTT TCC ATG G	58.3
FPL3328Fw	+	3328 - 3347	CCA TGT AGA CTA ACA CAT AC	55.1
FPL3328Rev	-	3328 - 3347	GTA TGT GTT AGT CTA CAT GG	55.1
FPL3863Fw	+	3863 - 3881	CTT ATG GTC CTT TAA CTG C	55.6
FPL3863Rev	-	3863 - 3881	GCA GTT AAA GGA CCA TAA G	55.6
FPL4001Fw	+	4001 - 4023	GTC AAT TAT TTG TAA AAG TTG CG	57.9
FPL4001Rev	-	4001 - 4023	CGC AAC TTT TAC AAA TAA TTG AC	57.9
FPL4234Rev	-	4234 - 4256	CTA GGT GCT AGT TGA GAT TTT TC	59.2

FPL4861badgerRev	-	4861 - 4885	CAC CAT AAC AAC ATA CAT TAA AAG	57.2
FPL4900Rev	-	4900 - 4923	CTT AAC ATA TTC TAA GGG CAA AC	57.9
FPL3180Fw **	+	3180 - 3204	TAC TGG AAC TAG TGG CAC ACC AAC	66
FPL3180Rev **	-	3180 - 3204	GTT GGT GTG CCA CTA GTT CCA GTA	66
FPL3740Fw **	+	3740 - 3765	ATC CAG AAG GAG ATT GGA TTC AAA A	62.9
FPL3740Rev **	-	3740 - 3765	TTT TGA ATC CAA TCT CCT TCT GGA T	62.9

* only used to sequence parvovirus in cats and dogs; **only used to sequence parvovirus in dogs. [17] Pérez, R.; Calleros, L.; Marandino, A.; Sarute, N.; Iraola, G.; Grecco, S.; Blanc, H.; Vignuzzi, M.; Isakov, O.; Shomron, N.; et al. Phylogenetic and Genome-Wide Deep-Sequencing Analyses of Canine Parvovirus Reveal Co-Infection with Field Variants and Emergence of a Recent Recombinant Strain. *PLoS One* **2014**, *9*, e111779, doi:10.1371/journal.pone.0111779.

Supplementary Table S2. GenBank Accession Numbers of the 21 parvoviral genomes used for designing the primers used in this study, including the species in which they have been classified by their researchers.

GenBank Acc. No.	Species	Host	Location
KP280068	FPV	Cat	China
KX685354	FPV	Tiger	China
MF069447	FPV	Raccoon	British Columbia
MG924893	FPV	Cat	China
MH559110	FPV	Cat	India
MK413737	FPV	Cat	Italy
MN862747	FPV	American mink	Newfoundland
MN862749	FPV	River otter	Newfoundland
MW811187	FPV	Cat	China
MZ005633	FPV	Lion	China
OM638043	FPV	Dog	Egypt
OM640096	FPV	Cat	Newfoundland
OQ266795	FPV	Cat	India
OR198066	FPV	Cat	Taiwan
OR227624	FPV	Cat	China
MN127781	FPV	Cat	Thailand
KX774252	CPV-2b	Dog	Brazil
AJ564427	CPV-2a	Dog	India
KU508407	CPV-2c	Dog	Italy
AY742934	CPV		Germany
EU659118	CPV	Dog	USA

Supplementary Table S3. Protocols of nested PCRs used for sequencing the complete genome of *Protoparvovirus carnivoran 1* from lynx (LG100), cat (LG15) and dog (LG151). The number in the name of the primer, where present, indicates the position in the genome of *Protoparvovirus carnivoran 1* sequenced from a badger in Italy (GenBank Acc. No. OP588004).

**Type of PCR**	**Primer combination**	**Expected Size of product (bp)**	**Annealing temp (°C)**
External 1	FPLutr1Fw or FPLutrgato1Fw + FPL1397Rev	≈1300	52
Internal 1.1	FPLutr2Fw or FPLutrgato2Fw + FPL937Rev	≈900	56
External 2	FPL28Fw + FPL2158Rev	2150	55
Internal 2.1	FPL438Fw + FPL1397Rev or FPL1699Rev	982 or 1282	52
Internal 2.2	FPL937Fw + FPL1699Rev	783	52
External 3	FPL937Fw + FPL3109Rev	2192	56
Internal 3.1	FPL1397Fw + FPL2158Rev	781	52
Internal 3.2	FPL1699Fw + FPL2509Rev	828	52
Internal 3.3	FPL2158Fw + FPL3109Rev	971	56
External 4	FPL1699Fw + FPL4234Rev	2557	52
Internal 4.1	FPL1699Fw + FPL3109Rev or FPL3180Rev	1430 or 1506	52 or 56
Internal 4.2	FPL2158Fw + FPL3180Rev	1047	56
Internal 4.3	FPL2384Fw + FPL3328Rev	965	51
Internal 4.4	FPL2509Fw + FPL3328Rev or FPL3740Rev	840 or 1256	51 or 54
Internal 4.5	FPL3109Fw or FPL3180Fw + FPL4001Rev	916 or 845	53
External 5	FPL2509Fw + FPL4900Rev	2415	53
Internal 5.1	FPL3328Fw + FPL4234Rev	930	51
Internal 5.2	FPL3863Fw + FPL4861badgerRev	1023	52

Supplementary Table S4. Amino acid comparison of VP2 region between strains from this study with other FPV and CPV-2 strains from NCBI. Sequences obtained in this study are shown in bold. VP2 amino acid sequences of stone marten (KP682526), Eurasian badger (KP682520) isolated in Spain [21], dog (OM638042), cat (KX434461) and Eurasian badger (OP588004) isolated in Italy, cat (MG924893) and jaguar (KX900570) isolated in China, are exactly like LG100lynx and are not included in the table. Likewise, VP2 amino acid sequences of dogs (MG013488, MT648203, MH476583, MW650830, MF805796, MN519258) isolated in China, in Vietnam (MT106233), and in Italy (OP588002), and in a Pangolin (MN832850) in Taiwan are exactly like LG151dog and are not included in the table.

Amino acid positioN (VP2)	5	6	7	9	13	16	19	21	29	37	52	58	66	67	70
LG100LYNX	A	V	Q	D	P	R	R	T	G	G	K	W	S	R	H
LG15CAT	.	.	.	.	.	.	.	.	.	.	.	.	.	.	.
LG151dog	G	.	.	.	.	.	.	.	.	.	.	.	.	.	.
MH559110	P	F	H	N	.	K	K	.	.	.	N	G	T	K	L
OQ266795	P	F	H	N	.	K	K	.	.	.	N	G	T	K	L
MW811187	.	.	.	.	.	.	.	.	.	.	.	.	.	.	.
KP280068	.	.	.	.	.	.	.	.	.	.	.	.	.	.	.
OR227624	.	.	.	.	.	.	.	.	.	.	.	.	.	.	.
KX685354	.	.	.	.	.	.	.	A	.	D	.	.	.	.	.
OR198066	.	.	.	.	.	.	.	.	.	.	.	.	.	.	.
MG764510	.	.	.	.	.	.	.	.	.	.	.	.	.	.	.
MZ044015	.	.	.	.	.	.	.	.	.	.	.	.	.	.	.
KR002793	.	.	.	.	.	.	.	.	.	.	.	.	.	.	.
OM721656	G	.	.	.	S	.	.	.	.	.	.	.	.	.	.
OP972595	.	.	.	.	.	.	.	.	.	.	.	.	.	.	.
OR230516	.	.	.	.	.	.	.	.	.	.	.	.	.	.	.
OP611195	G	.	.	.	.	.	.	.	.	.	.	.	.	.	.
MK388674	G	.	.	.	.	.	.	.	S	.	.	.	.	.	.

Amino acid position (VP2)	80	85	87	91	93	103	224	232	234	267	297	300	305	322	323
LG100lynx	K	N	M	A	K	V	G	V	H	F	S	A	D	T	D
LG15CAT	.	.	.	.	.	.	.	.	.	.	.	.	.	.	.
LG151dog	R	.	L	.	N	A	.	I	.	Y	A	G	Y	.	N
MH559110	.	.	.	.	.	.	.	.	.	.	.	.	.	.	.
OQ266795	.	.	.	.	.	.	.	.	.	.	.	.	.	.	.
MW811187	.	.	.	S	.	.	.	.	.	.	.	.	.	.	.
KP280068	.	.	.	.	.	.	.	.	.	.	.	.	.	.	.
OR227624	.	.	.	.	.	.	.	.	.	.	.	.	.	.	.
KX685354	.	.	.	.	.	.	.	.	.	.	.	.	.	.	.
OR198066	.	.	.	.	.	.	E	.	.	.	.	.	.	.	.
MG764510	.	I	.	.	.	.	.	I	.	.	.	.	.	.	.
MZ044015	.	.	.	.	.	.	.	.	Y	.	.	.	.	.	.
KR002793	R	.	L	.	N	A	.	I	.	Y	A	G	Y	.	N
OM721656	R	.	L	.	N	A	.	I	.	Y	A	G	Y	.	N
OP972595	R	.	L	.	N	A	.	I	.	Y	A	G	Y	A	N
OR230516	R	.	L	.	N	A	.	I	.	.	A	G	Y	.	N
OP611195	R	.	L	.	N	A	.	I	.	Y	A	G	Y	.	N
MK388674	R	.	L	.	N	A	.	I	.	Y	A	G	Y	.	N

Amino acid position (VP2)	324	370	373	390	412	426	440	447	564	568
LG100lynx	Y	Q	D	T	G	N	T	I	N	A

LG15cat	.	.	.	.	.	.	.	.	.	.
LG151dog	I	R	.	.	.	E	.	.	S	G
MH559110	.	.	N	A	R	.	.	.	.	.
OQ266795	.	.	N	A	R	.	.	.	.	.
MW811187	.	.	.	.	.	.	.	.	.	.
KP280068	.	.	.	.	.	.	.	.	.	.
OR227624	.	.	.	.	.	.	.	.	.	.
KX685354	.	.	.	.	.	.	.	.	.	.
OR198066	.	.	.	.	.	.	.	.	.	.
MG764510	.	.	.	.	.	.	.	.	.	.
MZ044015	.	.	.	.	.	.	.	.	.	.
KR002793	I	.	.	.	.	D	A	.	S	G
OM721656	I	.	.	.	.	D	A	.	S	G
OP972595	I	.	.	.	.	.	A	.	S	G
OR230516	L	.	.	.	.	D	.	.	S	G
OP611195	I	R	.	.	.	E	.	M	S	G
MK388674	I	R	.	.	.	E	.	.	S	G

Supplementary Table S5. GenBank Accession Numbers of the sequences used for constructing the phylogenetic tree of the VP2 protein, including the species in which they have been classified by their researchers. Only sequences enlisted in GenBank as complete genome were used for this construction.

GenBank Acc. No.	Species	Host	Location
AJ564427	CPV-2a	Dog	India
AY742934	CPV		Germany
EU659118	CPV	Dog	USA
KP280068	FPV	Cat	China
KP682520	FPV	Badger	Spain
KP682526	FPV	Stone marten	Spain
KP769859	FPV	Cat – brain	Belgium
KR002793	CPV-2b	Dog	China
KU508407	CPV-2c	Dog	Italy
KX434461	FPV	Cat	Italy
KX685354	FPV	Tiger	China
KX774252	CPV-2b	Dog	Brazil
KX900570	FPV	Jaguar	China
MF069447	FPV	Raccoon	British Columbia
MF805796	CPV	Dog	China
MG013488	CPV	Dog	China
MG764510	FPV	Tiger	China
MG764511	FPV	Panthera leo	China
MG924893	FPV	Cat	China
MH476583	CPV-2c	Dog	China
MH559110	FPV	Cat	India
MK388674	CPV-2c	Dog	China
MK413737	FPV	Cat	Italy
MN127781	FPV	Cat	Thailand
MN519258	CPV	Dog	China
MN832850	CPV-2c	Pangolin	Taiwan
MN862747	FPV	American mink	Newfoundland
MN862749	FPV	River otter	Newfoundland

MT106233	CPV-2c	Dog	Viet Nam
MT178243	FPV	Cheetah	China
MT614366	FPV	Cat	China
MT648203	CPV	Dog	China
MW650830	CPV	Dog	China
MW811187	FPV	Cat	China
MZ005633	FPV	Lion	China
MZ044015	AMD	Mink	China
MZ357119	FPV	Red panda	China
OM638042	FPV	Dog	Italy
OM638043	FPV	Dog	Egypt
OM640096	FPV	Cat	Newfoundland
OM721656	CPV-2b	Dog	Turkey
OP588002	CPV	Dog	Italy
OP588004	FPV	Badger	Italy
OP611195	CPV-2	Dog	Gabon
OP972595	CPV-2a	Dog	South Korea
OQ266795	FPV	Cat	India
OR198066	FPV	Cat	Taiwan
OR227624	FPV	Cat	China
OR230516	CPV-2a	Dog	Brazil
OR602717	FPV	Marsican brown bear	Italy
OR602718	FPV	Crested porcupine	Italy
PP781551 (LG100lynx)	FPV	Lynx	Spain
PQ436979 (LG15cat)	FPV	Cat	Spain
PQ436980 (LG151dog)	CPV-2c	Dog	Spain

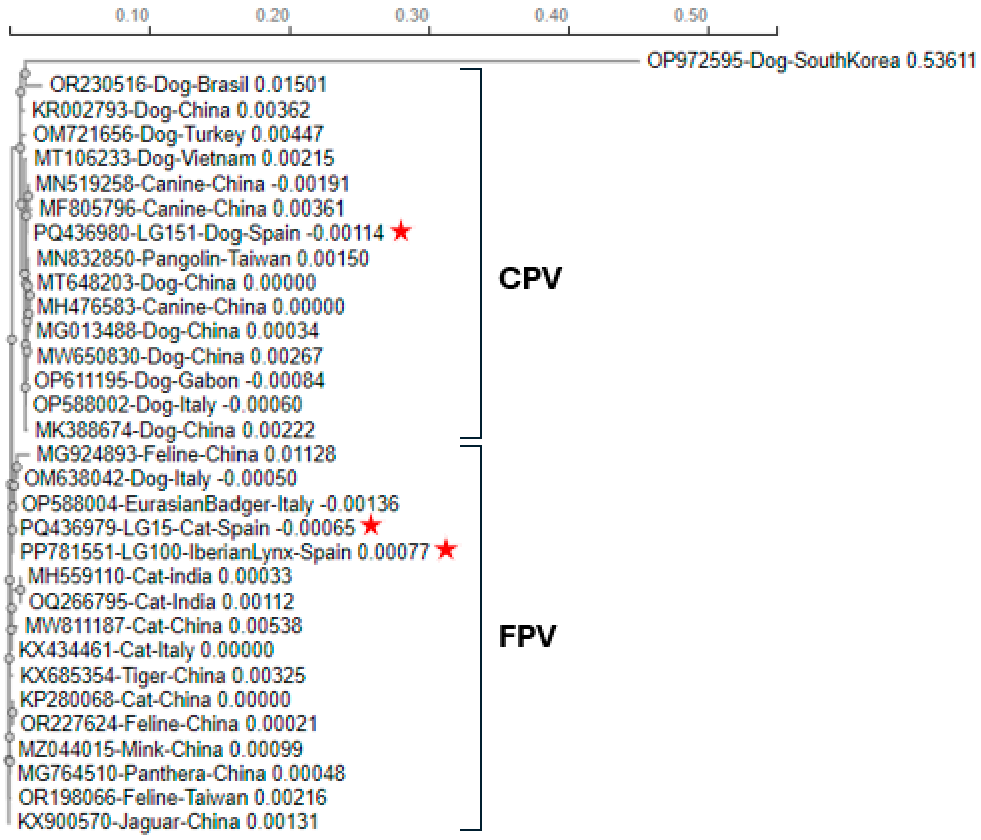

Supplementary Figure S1. Phylogenetic tree of the 32 genome sequences deposited in GenBank with which LG100lynx, LG15cat, and LG151dog (marked with red stars) were compared, including the species from which they were isolated and the geographic location.
